# Clinical Benefit of Tamsulosin and the Hexanic Extract of Serenoa Repens, in Combination or as Monotherapy, in Patients with Moderate/Severe LUTS-BPH: A Subset Analysis of the QUALIPROST Study

**DOI:** 10.3390/jcm9092909

**Published:** 2020-09-09

**Authors:** Antonio Alcaraz, Alfredo Rodríguez-Antolín, Joaquín Carballido-Rodríguez, David Castro-Díaz, Manuel Esteban-Fuertes, José M. Cózar-Olmo, Vincenzo Ficarra, Rafael Medina-López, Jesús M. Fernández-Gómez, Javier C. Angulo, José Medina-Polo, Francisco J. Brenes-Bermúdez, José M. Molero-García, Antonio Fernández-Pro-Ledesma, José Manasanch

**Affiliations:** 1 Urology Department, Hospital Clínic, Universitat de Barcelona, IDIBAPS, 08036 Barcelona, Spain; aalcaraz@clinic.cat; 2 Urology Department, Research Group in Men’s Integral Health, Instituto de Investigación i+12, Hospital Universitario 12 de Octubre, 28041 Madrid, Spain; arantolin@yahoo.es (A.R.-A.); josemedinapolo@movistar.es (J.M.-P.); 3 Urology Department, Hospital Universitario Puerta de Hierro Majadahonda, 28222 Majadahonda, Spain; carballidojoaquin@gmail.com; 4 Urology Department, Hospital Universitario de Canarias, 38320 Tenerife, Spain; davidmanuelcastrodiaz@gmail.com; 5 Urology Department, Hospital Nacional de Parapléjicos, 45004 Toledo, Spain; estebote@telefonica.net; 6 Urology Department, Hospital Universitario Virgen de las Nieves, 18014 Granada, Spain; cozarjm@yahoo.es; 7 Urology Department, University of Messina, 98125 Messina, Italy; vficarra@unime.it; 8 Urology Department, Hospital Universitario Virgen del Rocío, 41013 Sevilla, Spain; rantonio.medina.sspa@juntadeandalucia.es; 9 Urology Department, Hospital Universitario Central de Asturias, 33011 Oviedo, Spain; jmfergomez@gmail.com; 10 Urology Department, Hospital Universitario de Getafe, 28905 Getafe, Spain; jangulo1964@gmail.com; 11 ABS Llefià, 08913 Badalona, Spain; fjbrenesb@gmail.com; 12 San Andrés Primary Care Center, 28021 Madrid, Spain; jmolerog@gmail.com; 13 Menasalbas Primary Care Center, 45128 Toledo, Spain; afernandezprol@semg.es; 14 Pierre Fabre Ibérica S.A., 08005 Barcelona, Spain

**Keywords:** moderate-severe LUTS, BPH, BII, combination treatment, tamsulosin, hexanic extract of *Serenoa repens*, quality of life, storage symptoms, tolerability

## Abstract

To investigate whether tamsulosin (TAM) and the hexanic extract of *Serenoa repens* (HESr) are more effective in combination than as monotherapy in men with moderate-to-severe lower urinary tract symptoms associated with benign prostatic hyperplasia (LUTS/BPH). Subset analysis of data from a 6-month, multicenter observational study. Patients received either tamsulosin (0.4 mg/day) or HESr (320 mg/day) alone or in combination. Primary endpoints were change in symptoms and quality of life. Tolerability was also assessed. Seven hundred and nine patients were available for intention to treat (ITT) analysis, 263 treated with tamsulosin, 262 with HESr, and 184 with TAM + HESr. After 6 months, International Prostate Symptom Score (IPSS) scores improved by a mean (standard deviation) of 7.2 (5.0) points in the TAM + HESr group compared to 5.7 (4.3) points with TAM alone and 5.4 (4.6) points with HESr (*p* < 0.001). Quality of life showed greatest improvement with combination therapy (*p* < 0.02). Adverse effects were reported by 1.9% of patients receiving HESr, 13.3% receiving TAM, and 12.0% receiving TAM + HESr (*p* < 0.001). In men with moderate/severe LUTS/BPH, combination treatment with TAM + HESr produced more effective symptom relief and greater improvement in quality of life than with either treatment alone, with acceptable tolerability.

## 1. Introduction

Lower urinary tract symptoms (LUTS) are a common chronic condition in adult men and are frequently associated with benign prostatic hyperplasia (BPH), although they can also be caused by bladder dysfunction and other diseases affecting the urinary system [1]. The prevalence of LUTS increases with age [2], and the condition can have a marked effect on patient quality of life (QoL) [1] and the QoL of their partners [3], particularly when symptoms are moderate or severe (International Prostate Symptom Score (IPSS) > 7 points). The economic costs of LUTS/BPH (lower urinary tract symptoms associated with benign prostatic hyperplasia) are likewise considerable and expected to rise in the near future [4].

Commonly used medical treatments for the condition include alpha-adrenergic blockers (AB) and 5-alpha-reductase inhibitors (5ARI) which, while they may improve symptoms, have also been shown to negatively affect sexual function with studies showing that they can lead to problems, such as a reduction in libido, impotence, and/or ejaculatory problems [5], particularly when used in combination [6].

Another treatment for LUTS/BPH is the *Serenoa repens* extract, which has been shown to have anti-inflammatory [7,8,9], antiandrogenic [10,11], and antiproliferative effects [7,11]. Although several extracts of the plant are available commercially, there is considerable variation in potency across the different brands and type of extraction [7,12,13,14]. This variation in potency is reflected in the effectiveness of the different treatments, with some extracts of *S. repens* appearing to be no better than placebo [15,16]. On the other hand, a recent study (the Quality of Life in Benign Prostatic Hyperplasia, or QUALIPROST study) showed that, after 6 months of treatment in conditions of usual clinical practice, the hexanic extract of *Serenoa repens* (HESr) had a similar effect on symptoms and QoL as AB and 5ARI, with fewer adverse effects [17]. The efficacy and tolerability of the HESr has also been highlighted in two recent systematic reviews and meta-analysis [18,19]. On the basis of the existing evidence, a recent European Medicines Agency (EMA) report by the Committee on Herbal Medicinal Products (HMPC) concluded that only the hexanic extract of *S. repens* (HESr) can be considered to be supported by sufficient evidence to warrant a well-established use as a medicinal product with recognised efficacy and acceptable safety for the treatment of LUTS/BPH [7]. This recognition has been underlined in the most recent European Association of Urology (EAU) guidelines [20], which states that only the hexanic extract of *Serenoa repens* has been recommended for well-established use by the HMPC.

The use of combination therapy is well-established in LUTS/BPH, particularly in patients with moderate or severe symptoms, when monotherapy fails to provide the expected level of symptom relief or when there is an increased risk of progression [1]. Combined treatment with an alpha-blocker and a plant extract has been reported to represent 49.7% of all combination therapies used to manage LUTS/BPH in general practice [21]. While previous studies have reported on the experience of using HESr in combination with alpha-blockers [22,23], with positive results for the combination treatment over alpha-blocker monotherapy, no study to date has simultaneously evaluated and compared the combined therapy and both constituent treatments separately in conditions of real-world practice.

The aim of this subset analysis from the QUALIPROST study was to investigate whether tamsulosin (TAM) and HESr are more effective when used in combination or alone in men with moderate to severe LUTS/BPH managed in conditions of usual clinical practice. We also evaluated and compared the tolerability of the combination treatment and monotherapy.

## 2. Experimental Section

### 2.1. Patients and Study Design

Data for this analysis was from the QUALIPROST study (ISRCTN11815680), a longitudinal, prospective, observational, multicenter study to evaluate change in symptoms and QoL in patients with moderate to severe LUTS/BPH. Patients were followed up over a 6-month period in a urological setting [17]. The study conformed to the Strengthening the Reporting of Observational Studies in Epidemiology (STROBE) guidelines [24].

Patients were excluded from QUALIPROST if they had received drug treatment for LUTS/BPH in the 6 months prior to inclusion, if they had received any drug treatment with a known effect on LUTS/BPH symptoms at any time in the 4 weeks prior to inclusion, if they had other urinary disorders, or if they had previously undergone surgery of the lower urinary tract. For the present subset analysis, data from patients ≥ 40 years of age with a diagnosis of LUTS/BPH and an IPSS score of ≥12 were included. The selected patients were treated with TAM, at a dose of 0.4 mg/day, hexanic extract of *Serenoa repens* (HESr, 320 mg/day) or TAM plus HESr in combination treatment.

In order to maximize comparability between the treatment groups in this sub-analysis and to reduce the possibility of bias, an iterative procedure was used to match patients in the treatment groups over several rounds until they were homogenous in terms of baseline IPSS and Benign Prostatic Hyperplasia Impact (BII) scores, maximum urinary flow (Qmax), prostate-specific antigen (PSA), and prostate volume. This approach allows for unequal sample sizes and removes only the minimum number of individuals necessary to make groups comparable.

### 2.2. Study Variables

Key endpoints in this subset analysis were change in mean score on the International Prostate Symptom Score (IPSS) and change in QoL assessed using the Benign Prostatic Hyperplasia Impact Index (BII).

The IPSS has a total of 8 questions, seven of which are used to assess symptoms of LUTS/BPH, including storage symptoms (urgency, frequency, nocturia) and voiding symptoms (incomplete emptying, intermittency, weak stream and straining to void). The eighth item is used to assess QoL associated with the condition. The overall score on the IPSS ranges from 0 to 35 for the symptom items, with a higher score indicating more severe symptoms, and from 0 to 5 for the QoL item (item 8).

The BII is a self-administered questionnaire consisting of 4 questions measuring the impact of urinary symptoms on physical discomfort, worries about health, symptom bother, and interference with usual activities during the past month [25]. Items are answered using a Likert scale, with 4 or 5 response options per item and scores range from 0 (best QoL) to 13 (worst QoL). Both the IPSS and the BII questionnaires were self-completed by patients at baseline and at the 6-month follow-up visit.

Sociodemographic data collected at baseline included age, and weight and height, which were used to calculate body mass index (BMI). Data collected included date of onset of urinary symptoms, year of LUTS/BPH diagnosis, IPSS and BII scores, results from diagnostic tests when available (digital rectal exam, prostate volume, Qmax, urine analysis, serum analysis, PSA), treatment received (yes/no, alpha-blockers, 5-alpha-reductase inhibitors, phytotherapy, other), and information on co-morbidities and their treatment. Side effects associated with treatments were recorded at follow-up.

Treatment compliance was assessed using the validated Spanish version of the Haynes-Sackett questionnaire [26], which asks about (a) patients’ difficulty taking the medication and (b) the number of tablets they have taken in the previous month. Patients taking between 80% and 110% of the prescribed dose are considered to show good adherence.

QUALIPROST was performed as a real-world study reflecting current patient management, in which investigators could prescribe any of the commercially available medical treatments according to their usual practice. A range of treatments were prescribed, including monotherapy and combination treatments (further details can be found in the earlier manuscript).

### 2.3. Statistical Analysis

This analysis focused on patients scoring ≥ 12 points on the IPSS at baseline who received either HESr alone, TAM alone, or TAM + HESr. The analyses were performed for this sub-group of the QUALIPROST study as a whole and for two sub-groups defined by symptom severity (IPSS score of 12–19 and ≥20 points).

With a matched sample size of 248, 249, and 173 patients, for tamsulosin, HESr, and combined therapy, respectively, in the intention to treat (ITT) analysis, and, given the observed mean effect (standard deviation (SD)) on IPSS at the end of follow-up of 5.7 (4.3), 5.4 (4.6), and 7.2 (5.0) points for tamsulosin, HESr, and combined therapy, respectively, we estimated that the study would have a power of 93.3% to detect a statistically significant pairwise difference between treatment groups at alpha = 0.05.

ANOVA was used for between-group comparisons in each round to achieve matched samples in the HESr, TAM, and TAM + HESr groups and the iterative procedure was repeated until the p-value exceeded 0.1, indicating that there were no significant differences between the groups on baseline IPSS and BII scores, Qmax, PSA, and prostate volume. To determine the success of the matching procedure, pre- and post-matching baseline between-group differences on the IPSS and BII total scores, the IPSS voiding and storage sub-scales, and IPSS item 7 (nocturia) were assessed using ANOVA test for the overall sample, as well as for sub-groups defined by severity.

Change over time within the different treatment groups and between-group differences in the size of change on the IPSS and BII were assessed using the ANOVA test. Student’s *t*-test was used for pairwise treatment comparisons with a significance level of 0.017 to take into account multiple testing.

Changes on items 1–7 on the IPSS, which assess symptom severity, were analyzed separately from item 8, which assesses QoL. A responder analysis of patients was performed using results on IPSS items 1–7 and the results for the treatment groups compared using the chi-squared test or Fisher test as appropriate. Responders were defined as patients who improved by 3 points or more, a change which is considered clinically relevant [27]. The same analysis was repeated for patients showing an improvement of ≥25% in their IPSS score.

The correlation between scores on the BII score and scores on question 8 of the IPSS questionnaire was assessed using Pearson’s r. Side effects proportions were compared between the three treatment groups using a chi-squared or Fisher test as appropriate.

To further minimize the risk of bias, all analyses were carried out using intent to treat (ITT) and per protocol (PP) samples. In the PP approach, patients with any missing data on the IPSS and BII at any visit were excluded from the analysis as were any patients who were lost to follow-up. In all comparisons, results were considered statistically significant at *p* < 0.05. Statistical analyses were carried out using R 3.5.2 statistical software.

## 3. Results

**Figure 1:**
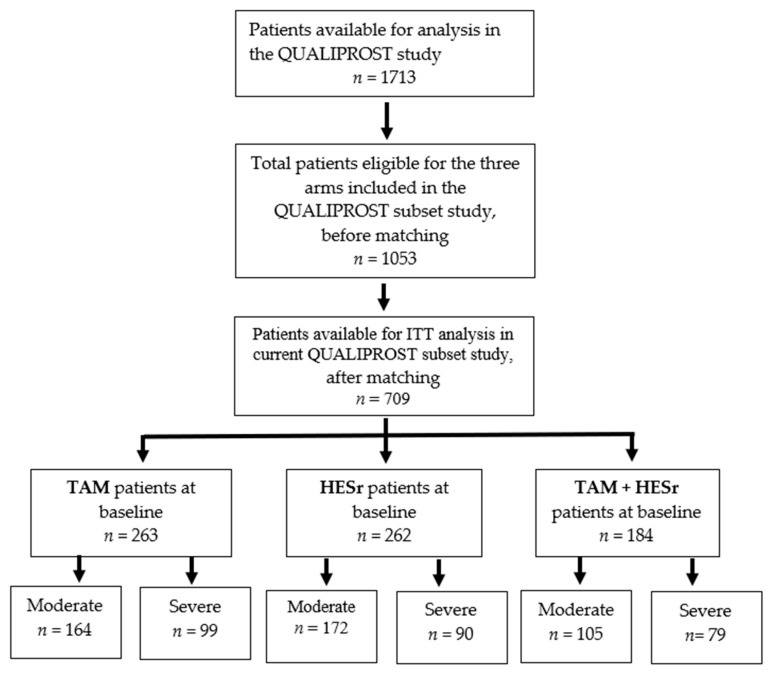
Study flow-chart.

After matching, a total of 709 patients were available for ITT analysis (*n* = 262 treated with HESr, *n* = 263 treated with TAM, and *n* = 184 treated with TAM + HESr). Figure 1 shows the study flow chart and the distribution of patients by treatment.

Table 1 shows the patient characteristics for the three treatment groups at baseline for the matched samples. There were no statistically significant differences between the treatment arms on any of the parameters analyzed. Similarly, there were no statistically significant differences between groups in the frequency of recorded concomitant diseases, except for a slightly higher incidence of dyslipidaemia in the combination treatment group (Table S1).

**Figure 2:**
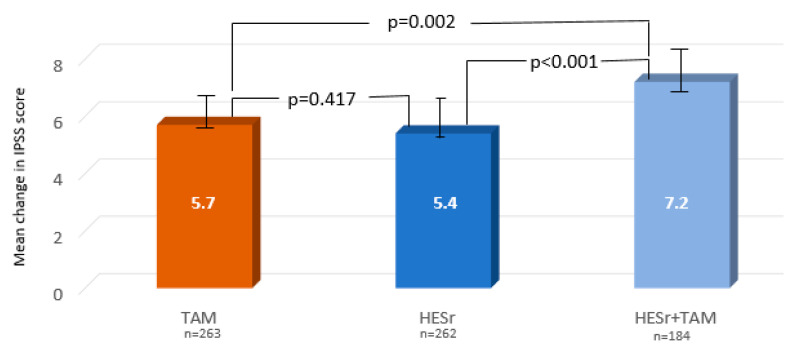
Mean improvement (95% confidence interval (CI)) in IPSS total score from baseline to 6 months for the three treatment groups (paired comparisons between the 3 groups, ITT analysis). IPSS: International Prostate Symptom Score; TAM: tamsulosin; HESr: hexanic extract of *Serenoa repens*. Student’s *t*-test.

Table 2 shows the change in symptoms and QoL for the three study groups after 6 months of treatment, based on ITT analysis. ANOVA showed statistically significant differences between the three treatment regimens (*p* < 0.001). By symptom type, the benefit of combination therapy over monotherapy was primarily in terms of the effect on storage symptoms. Nocturia, as measured by the specific question of the IPSS questionnaire, improved more in the combination group (mean (SD) change of 1.0 point (1.0)) than in either the TAM (mean (SD) change of 0.7 (0.9)) or HESr (0.8 (1.0)) groups, but the difference was only significant with TAM (*p* = 0.015). When change in IPSS total score was compared two-by-two among the three treatment strategies, we found that the combination treatment showed statistically significant better outcomes than either monotherapy (*p* = 0.002 and *p* < 0.001 versus tamsulosin and HESr, respectively), while no statistically significant differences between TAM and HESr were observed (Figure 2).

**Figure 3:**
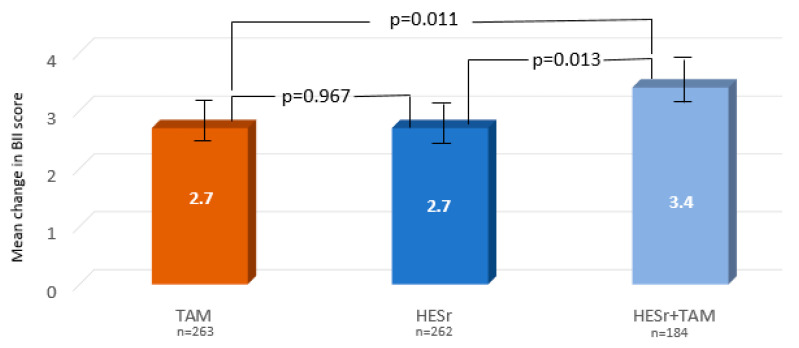
Mean improvement (95% confidence interval [CI]) in BII total score from baseline to 6 months. for the three treatment groups (paired comparisons between the 3 groups, ITT analysis). BII: BPH impact index; TAM: tamsulosin; HESr: hexanic extract of *Serenoa repens*. Student’s *t*-test.

The two QoL indicators (BII total score and IPSS item 8) also showed a statistically significant advantage for the combination therapy over either of the two monotherapies (*p* < 0.02 for both QoL measures) and no statistically significant differences between the two monotherapies in paired comparisons of the three treatment groups (Figure 3). Qmax increased in all three groups, with a mean (SD) change of 2.9 ml/s (3.8) for TAM (*n* = 37); 3.1 (4.2) for HESr (*n* = 49); and 2 (2.8) for TAM + HESr (*n* = 56) (*p* = 0.24).

When analyzing the subgroup of patients with more severe baseline symptoms (IPSS > 19), the overall pattern of results was similar to those for the sample as a whole, i.e., more favorable outcomes for the combination therapy than for either of the two monotherapies on both the IPSS symptom score, the IPSS QoL item, and on the BII (*p* < 0.001, Table 3). However, the improvements observed were larger in the more severe patients. For example, the more severe group improved by 10.1 points on the IPSS total score compared to 7.2 points in the sample as a whole. On the BII, the more severe group improved by 3.9 points compared to 3.4 points in the sample as a whole.

In the supplementary data, changes on the IPSS total score, the IPSS storage and voiding sub-scores, the BII, and item 8 of the IPSS are also shown for each treatment group in terms of percent change in score at 6 months. Results are provided for the overall sample and for the most severe group of patients (Tables S2 and S3, respectively).

Data on change in Qmax and PSA is also provided in the supplementary data for patients in the three study groups who underwent these tests at both baseline and the 6-month follow-up visit. Given that this was an observational study in which clinicians applied their usual criteria for requesting Qmax and PSA analysis, the available sample for both tests was significantly reduced at follow-up. Based on the available data, the mean improvement in Qmax ranged from 2 to 3.1 mL/s with no statistically significant differences in change values between the three study groups. Similarly, the change in PSA values between the study groups, available at follow-up, have shown no statistically significant differences. (Table S4).

Results of the IPSS responder analysis showed that 78.2% of patients in the TAM group improved by at least 3 points on the IPSS compared to 74.7% in the HESr arm and 85.0% in the TAM + HESr group (*p* = 0.04). The difference was primarily related to changes in the group of patients with more severe symptoms (IPSS > 19). When response was defined as a 25% or greater improvement from baseline on the IPSS, 56.9% of patients in the TAM monotherapy group, 58.6% in the HESr monotherapy group, and 74.0% in the TAM + HESr group met or exceeded the threshold (*p* = 0.001). There were no statistically significant differences between the HESr and TAM monotherapy groups on any of these analyses.

A statistically significant (*p* < 0.001) correlation of ≥0.53 was observed between the BII total score and question 8 of the IPSS in all groups. Relatively few patients reported any difficulty taking the medication (11% for TAM; 8.3% for HESr; and 10.1% for TAM + HESr; no statistically significant differences). Among those patients that did report difficulty taking the medication, over 82% nevertheless reported good treatment adherence, with no statistically significant differences between groups.

Table 4 shows the incidence of adverse effects (AE) for the three treatment groups. The rates of AE were highest in those receiving TAM and combination monotherapy (13.3% and 12% of patients reporting any adverse effect, respectively) and lowest in those receiving HESr as monotherapy (1.9%; *p* < 0.001). The most frequent AE was ejaculation disturbances, which was reported by 11.4%, 8.2% and 0.8% of patients receiving TAM, combination therapy, and HESr, respectively. Differences between the groups were statistically significant, favoring HESr (*p* < 0.001). Erectile dysfunction and hypotension also had a higher incidence in the TAM and TAM + HESr groups in comparison with the HESr group (*p* = 0.011).

## 4. Discussion

As far as we know, this is the first study to prospectively evaluate the efficacy and safety of TAM and HESr as monotherapy in the management of LUTS/BPH and to compare the results with those obtained using a combination of both treatments. Overall, this subset analysis from the QUALIPROST study showed that all three treatment options are effective in relieving LUTS/BPH, but that combination treatment is associated with greater improvement in symptoms and QoL than either TAM or HESr alone.

Given the potential for synergies between HESr’s demonstrated anti-inflammatory [1,7,8,9,28], antiandrogenic [10,11], and antiproliferative [11] effects and the relaxation of prostatic smooth muscle achieved with TAM [29], it is reasonable to expect that a combination of HESr and TAM would lead to greater symptom relief than could be obtained with either treatment individually. This hypothesis is further supported by the results of a study which found that storage symptoms improved significantly more with TAM + HESr vs TAM alone after 12 months of treatment [22], which may be due to the HESr’s anti-inflammatory action. In another study comparing the efficacy of silodosin versus silodosin plus HESr over a mean follow-up period of 13.5 months, combined therapy showed significantly more improvement from baseline on the IPSS score than silodosin alone, without any increase in adverse effects [23]. The proportion of patients showing clinically meaningful improvement (>3 IPSS points) was also higher for combination treatment (69.9% vs. 30.1% for silodosin alone; *p* = 0.001). As in our study, Boeri et al. also reported improvement in both storage and voiding symptoms and greater improvement in the most severe patients (IPSS > 19), which is the group in which combination treatment is most likely to be needed.

The improvements observed in the monotherapy arms in our study are similar to improvements reported in the PERMAL Study [30], a randomized controlled trial (RCT) comparing tamsulosin with the HESr in which both drugs reduced IPSS and Qmax to the same extent, though tolerability, in terms of impact on sexual function, was superior for the HESr. A subset analysis of this RCT focusing exclusively on severe LUTS/BPH patients (IPSS > 19) [31] also found equivalent improvements in IPSS (and its sub-scores) and QoL to the those observed in the HESr arm in our analysis. For example, improvements on total IPSS, storage IPSS, voiding IPSS, and IPSS question 8 (all units for these figures are points of the IPSS questionnaire) were 7.8 versus 7.8, 2.9 versus 3.2, 4.9 versus 4.6, and 1.2 versus 1.4 in the Debruyne et al. trial [31] compared to the present analysis, respectively.

The improvement of 7.2 points in mean IPSS score observed in our combination arm was also similar to the 6.4 point improvement reported for silodosin plus HESr [23] and to results reported in different RCTs which evaluated the effect of combining an alpha-blocker and a 5ARI to treat LUTS/BPH [6,32,33,34]. The enhanced effectiveness achieved with a combination of tamsulosin and HESr over the HESr alone has also been reported in a recent meta-analysis [19].

In relation to QoL, the improvement on overall BII score and on question 8 of the IPSS for the TAM and combination arms are in line with results reported in the CombAT study, which also used the BII questionnaire [35]. For example, we observed an improvement of 1.8 and 1.3 points in the combination and tamsulosin arms, respectively, compared to an improvement of 1.4 and 1.1 points for the same groups in the CombAT trial. Furthermore, the present analysis suggests that changes in symptoms are linked to modifications in QoL, as the pattern of improvement on the IPSS was similar to that seen on the BII, i.e., all three arms showed improvement in QoL, but the largest mean improvement was seen in patients receiving combination therapy (mean 3.4-point improvement in overall BII score compared to 2.7 in the monotherapy groups). Again, the change was largest in the most severe LUTS/BPH group receiving combination therapy (mean improvement of 3.9 points).

A clear advantage of the combination of TAM and HESr is that it has a very positive benefit/risk ratio. Twelve percent of patients in the combination arm reported adverse effects, which is very similar to the figure for the TAM arm (13.3%), though effectiveness was greater in the combination arm. This is a very favourable finding, especially when compared to the rate of 28% of drug-related adverse events reported for the combination of tamsulosin plus dutasteride, which is frequently employed to treat LUTS/BPH [6]. Such a combination mainly affects sexual function, which may lead to poorer patient QoL. For example, Rosen et al. [36] reported a negative, statistically significant difference between tamsulosin plus dutasteride and placebo in the sexual activity, sexual desire, and bother domains of the Men’s Sexual Health Questionnaire in a post hoc analysis of a double-blind, randomized study in sexually active patients. The negative impact on sexual function could also threaten treatment adherence if it leads to some patients stopping taking their treatment [37].

It was beyond the scope of the present study to investigate the potential effect of the TAM + HESr combination on BPH disease progression. Nevertheless, if progression is slow and limited in LUTS/BPH patients, as earlier studies in patients receiving placebo [34] and at more risk of progression [6], have shown, then using more aggressive treatment with a poorer adverse effect profile to try to slow progression may not be the optimal approach to patient management. The most appropriate strategy would appear to be to initially use the least aggressive option which has also demonstrated effectiveness. Combination treatment with TAM and HESr meets those criteria, as it substantially reduces symptom bother, has a smaller impact on sexual functioning compared to other combinations, and avoids the risk of acute coronary syndrome associated with 5ARIs [38]. Patients should of course be carefully monitored to ensure treatment provides sufficient symptom relief and only if such relief is not obtained, or if there is a real risk of disease progression, should consideration be given to moving to another treatment combination. The development of patient decision-making aids might be of use in this scenario.

The present study has some limitations. Data were obtained under conditions of real-world practice with no randomization or blinding; patients were therefore allocated to a specific management approach based on clinician judgment, which could lead to a selection bias. Nevertheless, the matching strategy used ensured that all groups included in this analysis were comparable at baseline on the most relevant study variables and should therefore have helped to minimise bias and enhance the robustness of the results. Furthermore, the results of the PP analysis confirmed those obtained in the ITT analysis. The absence of a placebo arm can also be considered a potential limitation. Nevertheless, as the study was intended to reflect current clinical practice, inclusion of a placebo arm would have potentially raised ethical issues and would not have been appropriate within the chosen given methodological approach. Finally, in relation to Qmax and PSA, the relatively small number of patients repeating the test at 6-month follow-up may have limited the possibility of observing statistically significant differences between the groups on this parameter. As this was an observational study, participating clinicians were not required to perform a flowmetry test or determination of PSA if they did not consider it necessary. This considerably reduced the number of patients in which we could assess change on these parameters and meant that we were unable to investigate the correlation between change on Qmax and PSA and change in symptoms and quality of life.

Strengths of the present study include the relatively large number of patients, the fact that outcomes were assessed in conditions of current clinical practice, and the matching approach used to ensure maximum comparability between the three treatment arms.

It would be of interest to perform longer-term study evaluating the efficacy of the three treatments used in this analysis on disease progression (i.e., on relative risk for acute urinary retention, BPH-related surgery, and BPH clinical progression) in the future.

## 5. Conclusions

In summary, as the origin of LUTS is multifactorial [20], combined medical treatment which includes drugs with different modes of action is of interest in clinical practice as it may offer advantages over specific treatments used as monotherapy [39]. This would be the case, for instance, with a combination of TAM and HESr. The role of inflammation as a potential new target for medical therapy of LUTS/BPH has also highlighted the relevance of plant extracts, such as HESr, which have been shown to have an anti-inflammatory action [40].

In the present study, combined treatment with TAM + HESr achieved larger improvements in IPSS and QoL scores than either of the two treatments used in monotherapy. The combination of TAM + HESr could be of interest for patients with LUTS/BPH who experience limited improvement with tamsulosin or the HESr alone and/or who are concerned about the impact of other combinations, such as an AB + 5RI, on sexual functioning. As noted above, management strategies should aim to maximize benefits while minimizing harm; medical treatments for LUTS/BPH therefore need to be tailored to the individual patient’s symptomatology, comorbidities, and preferences. The decision-making process should be based on accurate patient counseling in terms of the potential efficacy and tolerability of the different treatments available.

## Figures and Tables

**Table 1:** Patient characteristics at baseline for the three study groups (ITT analysis).

Variable	TAM		HESr		TAM + HESr		*p* Value
	*N* *	Mean (SD)	*N* *	Mean (SD)	*N* *	Mean (SD)	
Age (years)	238	65.4 (8.0)	223	64.6 (8.9)	161	65.1 (8.0)	0.589
BMI (Kg/m^2^)	233	26.9 (3.1)	221	26.8 (3.0)	160	27.1 (2.9)	0.514
IPSS total	263	18.7 (4.5)	262	18.6 (4.8)	184	19.5 (4.9)	0.150
BII	263	7.8 (2.1)	262	7.9 (1.9)	184	8.2 (2.1)	0.102
IPSS storage subscore	263	7.9 (2.1)	262	8.1 (2.2)	184	8.4 (2.2)	0.105
IPSS voiding subscore	263	10.8 (3.1)	262	10.5 (3.1)	184	11.1 (3.2)	0.209
IPSS item 8 (QoL)	263	3.9 (1.0)	262	3.8 (1.1)	184	4.0 (1.0)	0.372
Time since diagnosis (years)	237	1.1 (2.6)	222	1.2 (2.7)	159	1.4 (3.2)	0.607
Qmax (ml/s)	104	12.1 (3.7)	117	13.2 (4.1)	118	12.8 (3.1)	0.105
PSA (ng/ml)	240	2.5 (1.3)	242	2.3 (1.2)	176	2.4 (1.4)	0.409
Prostate volume (cm^3^)	220	52.3 (18.3)	230	51.1 (18.0)	169	54.8 (16.4)	0.106

HESr: hexanic extract of *Serenoa repens;* TAM: tamsulosin; BMI: body mass index; IPSS: International Prostate Symptom Score; BII: Benign Prostatic Hyperplasia Impact Index; ITT: intention to treat; SD: standard deviation. * Number of patients might vary according to the test and the personal clinical practice of the investigators.

**Table 2:** Improvement from baseline to 6-month follow-up in symptoms and quality of life scores for the three study groups, all patients (ANOVA, ITT analysis).

	TAM (*n* = 263)	HESr (*n* = 262)	TAM + HESr (*n* = 184)	*p* Value
IPSS total *	5.7 (4.3)	5.4 (4.6)	7.2 (5.0)	<0.001
BII total *	2.7 (2.4)	2.7 (2.5)	3.4 (2.5)	0.020
IPSS storage subscore *	2.4 (2.1)	2.4 (2.2)	3.2 (2.3)	<0.001
IPSS voiding subscore *	3.3 (2.8)	3.0 (3.0)	3.9 (3.3)	0.014
IPSS item 8 (QoL) *	1.3 (1.2)	1.3 (1.3)	1.8 (1.2)	<0.001

ITT: Intention to treat; HESr: hexanic extract of *Serenoa repens;* TAM: tamsulosin. IPSS: International Prostate Symptom Score; BII: Benign Prostatic Hyperplasia Impact Index. * Mean (standard deviation).

**Table 3:** Improvement from baseline to 6-month follow-up in symptoms and quality of life scores for the three study groups, in patients with severe (IPSS > 19) baseline symptoms (ANOVA, ITT analysis).

	TAM (*n* = 99)	HESr (*n* = 90)	TAM + HESr (*n* = 79)	*p* Value
IPSS total *	8.0 (5.0)	7.8 (5.1)	10.1 (5.1)	0.006
BII total *	2.9 (2.7)	2.9 (2.7)	3.9 (2.4)	0.021
IPSS storage subscore *	3.3 (2.3)	3.2 (2.4)	4.3 (2.4)	0.005
IPSS voiding subscore *	4.7 (3.2)	4.6 (3.2)	5.8 (3.2)	0.026
IPSS item 8 (QoL) *	1.6 (1.3)	1.4 (1.4)	2.2 (1.0)	<0.001

ITT: intention to treat; HESr: hexanic extract of *Serenoa repens;* TAM: tamsulosin. IPSS: International Prostate Symptom Score; BII: Benign Prostatic Hyperplasia Impact Index. * Mean (SD).

**Table 4:** Reported adverse effects (AE) by treatment group (ITT analysis). Only AE with an incidence of ≥1% in any of the groups are reported.

	TAM (*n* = 263)	HESr (*n* = 262)	TAM + HESr (*n* = 184)	*p* Value
Any adverse effect	35 (13.3%)	5 (1.2%)	22 (12.0%)	<0.001
Ejaculation disturbances *	30 (11.4%)	2 (0.8%)	15 (8.2%)	<0.001
Erectile dysfunction	3 (1.1%)	0 (0.0%)	5 (2.7%)	0.021
Hypotension	7 (2.7%)	0 (0.0%)	5 (2.7%)	0.011
Reduced libido	3 (1.1%)	0 (0.0%)	1 (0.5%)	0.198
Dizziness	0 (0.0%)	2 (0.8%)	2 (1.1%)	0.253

ITT: intention to treat. * Ejaculation disturbances include retrograde ejaculation and decreased ejaculation volume. The PP analysis was carried out in a sample of 603 patients with complete IPSS and BII data who reported good treatment adherence and for whom data was available at baseline and the 6-month visit. The same pattern of results was observed as in the ITT group, with no relevant differences between the two sets of analysis. Results are shown as supplementary data (Tables S5–S8 and Figures S1–S3).

## Supplementary Materials

The following are available online at https://www.mdpi.com/2077-0383/9/9/2909/s1. Figure S1: Study flow-chart (PP sample), Figure S2: Mean improvement (95% CI) in IPSS total score from baseline to 6 months for the three treatment groups (PP analysis), Figure S3: Mean improvement (95% CI) in BII total score from baseline to 6 months for the three treatment groups (PP analysis), Table S1: Concomitant diseases at baseline for the three study groups, *n* (%), Table S2: Percent change in symptoms and quality of life scores from baseline to 6-month follow-up in the three study groups, all patients (ANOVA, ITT analysis), Table S3: Percent change in symptoms and quality of life scores from baseline to 6-month follow-up in the three study groups for patients with severe (IPSS > 19) baseline symptoms (ANOVA, ITT analysis), Table S4: Change from baseline to 6-month follow-up in Qmax and PSA for the three study groups, all patients (ANOVA, ITT analysis). Table S5: Patient characteristics at baseline for the three study groups (PP analysis), Table S6: Change from baseline to 6-month follow-up in symptoms and quality of life for the three study groups, all patients (ANOVA, PP analysis), Table S7: Change from baseline to 6-month follow-up in symptoms and quality of life scores for the three study groups in patients with severe (IPSS > 19) baseline symptoms (ANOVA, PP analysis) Table S8: Change from baseline to 6-month follow-up in Qmax and PSA for the three study groups, all patients (ANOVA, PP analysis).

## Appendix A

The following investigators participated in the QUALIPROST Study Group:

A *Coruña*: Dr. L. Busto Castañón; Dr. J.E. Duarte Novo; Dr. M. Montes Couceiro; Dr. A. Rodríguez Alonso; *Alicante*: Dr. J.A. Canovas Ivorra; Dr. C. López López; Dr. L. Prieto Chaparro; *Almería*: Dr. F. Gómez Berjón; Dr. J. Hortelano Parras; *Asturias*: Dr. A. M. Huescar; Dr. P.L. Muntañola Armora; *Badajoz*: Dr. E. Godoy Rubio; Dr. J. Mariño del Real; *Barcelona*: Dr. J. Armona Mani; Dr. F.J. Blasco Casares; Dr. S. Bucar Terrades; Dr. Ll. Cortadellas Angel; Dr. J. Fernández Zuazu; Dr. J.M. Malet Carreras; Dr. S. Mando Dakkar; Dr. J.M. Prats Puig; Dr. M. Puyol Pallás; Dr. J.A. Romero Martín; Dr. J. Sáenz de Cabezón Martí; Dr. J. Sánchez Macías; Dr. C. Vargas Blasco; Dr. J.M. Vayreda Martija; *Bilbao*: Dr. J.A. Gallego Sánchez; Dr. N. Prieto Ugidos; *Cádiz*: Dr. J.M. Arroyo Maestre; Dr. S. Garrido Insua; Dr. A. Gutiérrez de Pablo; Dr. M. Marmol Ruíz; Dr. F. Reyes Martínez; *Castellón*: Dr. J. Beltrán Persiva; Dr. L. Monzonis Rey; Dr. J. Palacios Castañeda; *Ciudad Real*: Dr. N. Jiménez López-Lucendo; Dr. D. Rodríguez Leal; *Córdoba*: Dr. J.C. Regueiro López; *Granada*: Dr. M. Cabezas Zamora; *Guipúzcoa*: Dr. G. Garmendia Olaizola; Dr. J.A. Rodríguez Andrés; *Jaén*: Dr. J. Jiménez Verdejo; Dr. E.J. Zarate Rodríguez; *León*: Dr. S.C. Gómez Cisneros; Dr. M. Lozano Rebollo; *Lleida*: Dr. J. Cortada Robert; Dr. L.M. Flavian Domenech; *Logroño*: Dr. A. Fernández Fernández; *Lugo*: Dr. F.J. Neira Pampin; *Madrid*: Dr. A. Abdallah Merhi; Dr. F. Arias Funes; Dr. I.T. Castillón Vela; Dr. J.M. Duarte Ojeda; Dr. M.J. García-Matres Cortés; Dr. J.F. Hermina Gutierrez; Dr. J.J. López-Tello García; Dr. C. Martín García; Dr. B. M.El-Awadeh; Dr. J.D. Rendón Sánchez; Dr. R. Rodríguez-Patron Rodríguez; Dr. J.C. Ruíz de la Roja; Dr. J. Vallejo Herrador; Dr. D. Vázquez Alba; *Málaga*: Dr. A. Bonilla Maldonado; Dr. J.M. Fernández Montero; Dr. A. Galacho Bech; Dr. C. Marchal Escalona; Dr. P. Rodero García; Dr. G. Sanz; Dr. J.D. Vázquez Cervilla; *Murcia*: Dr. G. Server Pastor; *Pontevedra*: Dr. D. Jamardo González; Dr. A. Selas Pérez; *Salamanca*: Dr. F. Díaz Alférez; Dr. M.F. Lorenzo Gómez; *Santander*: Dr. J.A. Portillo Martín; *Sevilla*: Dr. F.J. Giráldez Puig; Dr. M.A. Gutiérrez González; Dr. J.I. Huesa Martínez; Dr. Y. Ismail Tomaizeh; Dr. J. Leal Arenas; Dr. J. Martín Calero; Dr. J.L. Moyano Calvo; Dr. A. Ortiz Gámiz; Dr. J.M. Poyato Galán; Dr. J.A. Valero Puerta; Dr. E. Vilches Cocovi; *Tarragona*: Dr. P.L. Álvarez de la Red; Dr. J. Benagues Pamies; Dr. M. Prados Saavedra; *Tenerife*: Dr. T. Concepción Masip; Dr. C. Gómez de Segura Melcon; Dr. E. González de Chaves; Dr. J. Postius Robert; *Toledo*: Dr. F. Álvarez Fernández; Dr. A. Iglesias Justo; Dr. A. Melchor Galán; *Valencia*: Dr. J.E. Blasco Alfonso; Dr. M.A. Bonillo García; Dr. A. Collado Serra; Dr. J.A. García Cebrián; Dr. L.García Reboll; Dr. Y. Pallás Costa; Dr. M.J. Sánchez Sanchiz; Dr. J. Santamaría Meseguer; *Zaragoza*: Dr. A. García de Jalón Martínez; Dr. J.M. Gil Fabra; Dr. F. Monzón Alebesque; Dr. A. Ucar Terren.

## References

[1] Gravas S., Cornu J.N., Gacci M., Gratzke C., Herrmann T.R.W., Mamoulakis C., Rieken M., Speakman M.J., Tikkinen K.A.O. (2019). Management of Non-Neurogenic Male Lower Urinary Tract Symptoms (LUTS), Incl. Benign Prostatic Obstruction (BPO).

[2] Fitzpatrick J.M. (2006). The natural history of benign prostatic hyperplasia. BJU Int..

[3] Speakman M., Kirby R., Doyle S., Ioannou C. (2015). Burden of male lower urinary tract symptoms (LUTS) suggestive of benign prostatic hyperplasia (BPH)—focus on the UK. BJU Int..

[4] Vuichoud C., Loughlin K.R. (2015). Benign prostatic hyperplasia: Epidemiology, economics and evaluation. Can. J. Urol..

[5] Gacci M., Ficarra V., Sebastianelli A., Corona G., Serni S., Shariat S.F., Maggi M., Zattoni F., Carini M., Novara G. (2014). Impact of medical treatments for male lower urinary tract symptoms due to benign prostatic hyperplasia on ejaculatory function: A systematic review and meta-analysis. J. Sex. Med..

[6] Roehrborn C.G., Siami P., Barkin J., Damião R., Major-Walker K., Nandy I., Morrill B.B., Gagnier R.P., Montorsi F., CombAT Study Group (2010). The effects of combination therapy with dutasteride and tamsulosin on clinical outcomes in men with symptomatic benign prostatic hyperplasia: 4-year results from the CombAT study. Eur. Urol..

[7] European Medicines Agency (2015). Assessment Report on Serenoa Repens (W. Bartram) Small, Fructus.

[8] Sirab N., Robert G., Fasolo V., Descazeaud A., Vacherot F., Taille A.D., Terry S. (2013). Lipidosterolic extract of *Serenoa repens* modulates the expression of inflammation related-genes in benign prostatic hyperplasia epithelial and stromal cells. Int. J. Mol. Sci..

[9] Latil A., Libon C., Templier M., Junquero D., Lantoine-Adam F., Nguyen T. (2012). Hexanic lipidosterolic extract of *Serenoa repens* inhibits the expression of two key inflammatory mediators, MCP-1/CCL2 and VCAM-1, in vitro. BJU Int..

[10] Bayne C.W., Donnelly F., Ross M., Habib F.K. (1999). *Serenoa repens* (Permixon): A 5alpha-reductase types I and II inhibitor-new evidence in a coculture model of BPH. Prostate.

[11] Di Silverio F., Monti S., Sciarra A., Varasano P.A., Martini C., Lanzara S., D’Eramo G., di Nicola S., Toscano V. (1998). Effects of long-term treatment with *Serenoa repens* (Permixon) on the concentrations and regional distribution of androgens and epidermal growth factor in benign prostatic hyperplasia. Prostate.

[12] Scaglione F., Lucini V., Pannacci M., Dugnani S., Leone C. (2012). Comparison of the potency of 10 different brands of *Serenoa repens* extracts. Eur. Rev. Med. Pharmacol. Sci..

[13] Habib F.K., Wyllie M.G. (2004). Not all brands are created equal: A comparison of selected components of different brands of *Serenoa repens* extract. Prostate Cancer Prostatic Dis..

[14] Scaglione F., Lucini V., Pannacci M., Dugnani S., Leone C. (2008). Comparison of the potency of different brands of *Serenoa repens* extract on 5alpha-reductase types I and II in prostatic co-cultured epithelial and fibroblast cells. Pharmacology.

[15] Bent S., Kane C., Shinohara K., Neuhaus J., Hudes E.S., Goldberg H., Avins A.L. (2006). Saw palmetto for benign prostatic hyperplasia. N. Engl. J. Med..

[16] MacDonald R., Tacklind J.W., Rutks I., Wilt T.J. (2012). *Serenoa repens* monotherapy for benign prostatic hyperplasia (BPH): An updated Cochrane systematic review. BJU Int..

[17] Alcaraz A., Rodriguez J.C., Unda-Urzaiz M., Medina-Lopez R., Ruiz-Cerdá J.L., Rodríguez-Rubio F., García-Rojo D., Brenes-Bermúdez F.J., Cózar-Olmo J.M., Baena-González V. (2016). Quality of life in patients with lower urinary tract symptoms associated with BPH: Change over time in real-life practice according to treatment–the QUALIPROST study. Int. Urol. Nephrol..

[18] Vela-Navarrete R., Alcaraz A., Rodríguez-Antolín A., López B.M., Fernández-Gómez J.M., Angulo J.C., Diaz D.C., Romero-Otero J., Brenes F.J., Carballido J. (2018). Efficacy and safety of a hexanic extract of *Serenoa repens* (Permixon^®^) for the treatment of lower urinary tract symptoms associated with benign prostatic hyperplasia (LUTS/BPH): Systematic review and meta-analysis of randomized controlled trials and observational studies. BJU Int..

[19] Novara G., Giannarini G., Alcaraz A., Cózar-Olmo J.M., Descazeaud A., Montorsi F., Ficarra V. (2016). Efficacy and Safety of Hexanic Lipidosterolic Extract of *Serenoa Repens* (Permixon) in the Treatment of Lower Urinary Tract Symptoms Due to Benign Prostatic Hyperplasia: Systematic Review and Meta-analysis of Randomized Controlled Trials. Eur. Urol. Focus.

[20] Gravas S., Cornu J.N., Drake M.J., Gacci M., Gratzke C., Herrmann T.R.W., Tikkine K. EAU Guidelines on management of non-neurogenic male LUTS. Proceedings of the EAU Annual Congress Amsterdam 2020.

[21] Fourcade R.O., Lacoin F., Rouprêt M., Slama A., Le Fur C., Michel E., Sitbon A., Cotté F.E. (2012). Outcomes and general health-related quality of life among patients medically treated in general daily practice for lower urinary tract symptoms due to benign prostatic hyperplasia. World J. Urol..

[22] Ryu Y.W., Lim S.W., Kim J.H., Ahn S.H., Choi J.D. (2015). Comparison of tamsulosin plus *Serenoa repens* with tamsulosin in the treatment of benign prostatic hyperplasia in Korean men: 1-year randomized open label study. Urol. Int..

[23] Boeri L., Capogrosso P., Ventimiglia E., Cazzaniga W., Pederzoli F., Moretti D., Dehò F., Montanari E., Montorsi F., Salonia A. (2017). Clinically Meaningful Improvements in LUTS/BPH Severity in Men Treated with Silodosin Plus Hexanic Extract of *Serenoa repens* or Silodosin Alone. Sci. Rep..

[24] Von Elm E., Altman D.G., Egger M., Pocock S.J., Gøtzsche P.C., Vandenbroucke J.P. (2007). STROBE Initiative. Strengthening the Reporting of Observational Studies in Epidemiology (STROBE) statement: Guidelines for reporting observational studies. BMJ.

[25] Barry M.J., Fowler F.J., O’Leary M.P., Bruskewitz R.C., Holtgrewe H.L., Mebust W.K. (1995). Measuring disease-specific health status in men with benign prostatic hyperplasia. Measurement Committee of The American Urological Association. Med. Care.

[26] Haynes R.B., Sackett D.L., Gibson E.S., Taylor D.W., Hackett B.C., Roberts R.S., Johnson A.L. (1976). Improvement of medication compliance in uncontrolled hypertension. Lancet.

[27] Barry M.J., Williford W.O., Chang Y., Machi M., Jones K.M., Walker-Corkery E., Lepor H. (1995). Benign prostatic hyperplasia specific health status measures in clinical research: How much change in the American Urological Association symptom index and the benign prostatic hyperplasia impact index is perceptible to patients?. J. Urol..

[28] Ficarra V., Rossanese M., Zazzara M., Giannarini G., Abbinante M., Bartoletti R., Mirone V., Scaglione F. (2014). The role of inflammation in lower urinary tract symptoms (LUTS) due to benign prostatic hyperplasia (BPH) and its potential impact on medical therapy. Curr. Urol. Rep..

[29] Michel M.C. (2010). The forefront for novel therapeutic agents based on the pathophysiology of lower urinary tract dysfunction: Alpha-blockers in the treatment of male voiding dysfunction—how do they work and why do they differ in tolerability?. J. Pharmacol. Sci..

[30] Debruyne F., Koch G., Boyle P., da Silva F.C., Gillenwater J.G., Hamdy F.C., Perrin P., Teillac P., Vela-Navarrete R., Raynaud J.P. (2002). Comparison of a phytotherapeutic agent (Permixon) with an alpha-blocker (Tamsulosin) in the treatment of benign prostatic hyperplasia: A 1-year randomized international study. Eur. Urol..

[31] Debruyne F., Boyle P., Calais da Silva F., Gillenwater J.G., Hamdy F.C., Perrin P., Teillac P., Vela-Navarrete R., Raynaud J.P., Schulman C.C. (2004). Evaluation of the clinical benefit of Permixon and tamsulosin in severe BPH patients—PERMAL study subset analysis. Eur. Urol..

[32] Chung B.H., Roehrborn C.G., Siami P., Major-Walker K., Morrill B.B., Wilson T.H., Montorsi F. (2009). Efficacy and safety of dutasteride, tamsulosin and their combination in a subpopulation of the CombAT study: 2-year results in Asian men with moderate-to-severe BPH. Prostate Cancer Prostatic Dis..

[33] Lepor H., Williford W.O., Barry M.J., Brawer M.K., Dixon C.M., Gormley G., Haakenson C., Machi M., Narayan P., Padley R.J. (1996). The efficacy of terazosin, finasteride, or both in benign prostatic hyperplasia. Veterans Affairs Cooperative Studies Benign Prostatic Hyperplasia Study Group. N. Engl. J. Med..

[34] McConnell J.D., Roehrborn C.G., Bautista O.M., Andriole G.L., Dixon C.M., Kusek J.W., Lepor H., McVary K.T., Nyberg L.M., Clarke H.S. (2003). The long-term effect of doxazosin, finasteride, and combination therapy on the clinical progression of benign prostatic hyperplasia. N. Engl. J. Med..

[35] Barkin J., Roehrborn C.G., Siami P., Haillot O., Morrill B., Black L., Montorsi F., CombAT Study Group (2009). Effect of dutasteride, tamsulosin and the combination on patient-reported quality of life and treatment satisfaction in men with moderate-to-severe benign prostatic hyperplasia: 2-year data from the CombAT trial. BJU Int..

[36] Rosen R.C., Roehrborn C.G., Manyak M.J., Palacios-Moreno J.M., Wilson T.H., Lulic Z., Giuliano F. (2019). Evaluation of the impact of dutasteride/tamsulosin combination therapy on libido in sexually active men with lower urinary tract symptoms (LUTS) secondary to benign prostatic hyperplasia (BPH): A post hoc analysis of a prospective randomised placebo-controlled study. Int. J. Clin. Pract..

[37] Cindolo L., Pirozzi L., Fanizza C., Romero M., Tubaro A., Autorino R., de Nunzio C., Schips L. (2015). Drug adherence and clinical outcomes for patients under pharmacological therapy for lower urinary tract symptoms related to benign prostatic hyperplasia: Population-based cohort study. Eur. Urol..

[38] Chou C.H., Lin C.L., Lin M.C., Sung F.C., Kao C.H. (2015). 5α-Reductase inhibitors increase acute coronary syndrome risk in patients with benign prostate hyperplasia. J. Endocrinol. Investig..

[39] Serati M., Andersson K.E., Dmochowski R., Agrò E.F., Heesakkers J., Iacovelli V., Novara G., Khullar V., Chapple C. (2019). Systematic Review of Combination Drug Therapy for Non-neurogenic Lower Urinary Tract Symptoms. Eur. Urol..

[40] De Nunzio C., Salonia A., Gacci M., Ficarra V. (2020). Inflammation is a target of medical treatment for lower urinary tract symptoms associated with benign prostatic hyperplasia. World J. Urol..

