# Supplementary material for: Clinical Benefit of Tamsulosin and the Hexanic Extract of Serenoa Repens, in Combination or as Monotherapy, in Patients with Moderate/Severe LUTS-BPH: A Subset Analysis of the QUALIPROST Study
Source: J Clin Med. 2020 Sep 9;9(9):2909. doi: 10.3390/jcm9092909 (PMC7564885; doi:10.3390/jcm9092909)
Supplement: Supplementary file 1 [file jcm-09-02909-s001.pdf]

Supplementary Materials:

Table S1. Concomitant diseases at baseline for the three study groups, n (%).

**Concomitant disease**	**TAM** (n = 263)	**HESr** (n = 262)	**TAM + HESr** (n = 184)	**p value**
**Patients with any concomitant disease**	109 (41.4)	87 (33.2)	95 (51.6)	0.001
**Arterial hypertension**	63 (24.0)	53 (20.2)	54 (29.3)	0.085
**Dyslipidaemia**	46 (17.5)	38 (14.5)	50 (27.2)	0.003
**Diabetes mellitus**	33 (12.5)	27 (10.3)	30 (16.3)	0.172
**Other**	6 (2.3)	6 (2.3)	6 (3.3)	0.791

HESr*:* hexanic extract of *Serenoa repens;* TAM: tamsulosin.

Table S2. Percent change in symptoms and quality of life scores from baseline to 6-month follow-up in the three study groups, all patients (ANOVA, ITT analysis).

	**TAM** (n = 263)	**HESr** (n = 262)	**TAM + HESr** (n = 184)	**p value**
**IPSS total***	30.5	29.0	36.9	<0.001
**BII total***	34.6	29.0	36.9	0.020
**IPSS storage sub-score***	30.4	29.6	38.1	<0.001
**IPSS voiding sub-score***	30.6	28.6	35.1	0.014
**IPSS item 8 (QoL)***	33.3	34.2	45.0	<0.001

ITT: intention to treat; HESr*:* hexanic extract of *Serenoa repens;* TAM: tamsulosin;
IPSS: International Prostate Symptom Score; BII: Benign Prostatic Hyperplasia Impact Index
*data in percentage (%)

Table S3. Percent change in symptoms and quality of life scores from baseline to 6-month follow-up in the three study groups for patients with severe (IPSS > 19) baseline symptoms (ANOVA, ITT analysis).

	**TAM** (n = 99)	**HESr** (n = 90)	**TAM + HESr** (n = 79)	**p value**
**IPSS total***	34.2	32.5	42.1	0.006
**BII total***	33.3	34.5	43.3	0.021
**IPSS storage sub-score***	34.4	31.7	42.6	0.005
**IPSS voiding sub-score***	34.1	33.1	41.7	0.026
**IPSS item 8 (QoL)***	36.4	31.8	50.0	<0.001

ITT: Intention to treat; HESr*:* hexanic extract of *Serenoa repens;* TAM: tamsulosin;
IPSS: International Prostate Symptom Score; BII: Benign Prostatic Hyperplasia Impact Index
* data in percentage (%)

Table S4. Change from baseline to 6-month follow-up in Qmax and PSA for the three study groups, all patients (ANOVA, ITT analysis).

	TAM		HESr		TAM + HESr		p value
	n*	mean (SD)	n*	mean (SD)	n*	mean (SD)	
**Qmax (ml/s)**	37	2.9 (3.8)	49	3.1 (4.2)	56	2.0 (2.8)	0.238
**PSA (ng/ml)**	86	0.1 (1.5)	81	-0.1 (0.7)	79	-0.4 (1.1)	0.078

ITT: Intention to treat; HESr: hexanic extract of *Serenoa repens;* TAM: tamsulosin.
Qmax: maximum urinary flow; PSA: prostate-specific antigen
* Number of patients vary according to the test and the personal clinical practice of the investigators.

Table S5. Patient characteristics at baseline for the three study groups (PP analysis).

Variable	TAM		HESr		TAM + HESr		p value
	n*	mean (SD)	n*	mean (SD)	n*	mean (SD)	
**Age (years)**	201	65.8 (8.2)	187	64.7 (9.3)	142	65.1 (8.3)	0.482
**BMI (Kg/m^2)**	197	27.0 (3.1)	185	26.7 (3.0)	141	27.1 (2.9)	0.434
**IPSS total**	222	18.7 (4.4)	222	18.7 (4.7)	159	19.6 (4.9)	0.137
**BII**	222	7.8 (2.0)	222	7.9 (2.0)	159	8.3 (2.1)	0.112
**IPSS storage sub-score**	222	8.0 (2.1)	222	8.1 (2.2)	159	8.4 (2.2)	0.106
**IPSS voiding sub-score**	222	10.7 (3.1)	222	10.5 (3.1)	159	11.1 (3.2)	0.202
**IPSS item 8 (QoL)**	222	3.9 (0.9)	222	3.8 (1.1)	159	3.9 (1.0)	0.491
**Time since diagnosis (years)**	201	1.3 (2.7)	186	1.3 (2.9)	140	1.6 (3.4)	0.579
**Qmax (ml/s)**	86	12.2 (3.9)	97	13.3 (3.9)	98	13.2 (2.9)	0.111
**PSA (ng/ml)**	201	2.4 (1.2)	206	2.3 (1.2)	151	2.4 (1.4)	0.775
**Prostate volume (cm^3)**	192	52.5 (17.8)	197	51.2 (18.2)	147	55.1 (15.9)	0.106

HESr: hexanic extract of *Serenoa repens;* TAM: tamsulosin; BMI: body mass index; IPSS: International Prostate Symptom Score; BII: Benign Prostatic Hyperplasia Impact Index; PP: per protocol.
* Number of patients might vary according to the test and the current clinical practice of the investigators.

Table S6. Change from baseline to 6-month follow-up in symptoms and quality of life for the three study groups, all patients (ANOVA, PP analysis).

	TAM (n = 222)	HESr (n = 222)	TAM + HESr (n = 159)	p value
**IPSS total***	5.9 (4.2)	5.6 (4.6)	7.3 (5.1)	0.002
**BII total***	2.8 (2.3)	2.6 (2.6)	3.4 (2.5)	0.007
**IPSS storage sub-score***	2.5 (2.1)	2.5 (2.2)	3.3 (2.4)	<0.001
**IPSS voiding sub-score***	3.4 (2.8)	3.1 (3.0)	4.1 (3.4)	0.016
**IPSS item 8 (QoL)***	1.4 (1.2)	1.3 (1.3)	1.8 (1.2)	<0.001

PP: per protocol; HESr: hexanic extract of *Serenoa repens;* TAM: tamsulosin;
IPSS: International Prostate Symptom Score; BII: Benign Prostatic Hyperplasia Impact Index
* Mean (SD)

Table S7. Change from baseline to 6-month follow-up in symptoms and quality of life scores for the three study groups in patients with severe (IPSS > 19) baseline symptoms (ANOVA, PP analysis).

	**TAM** (n = 82)	**HESr** (n = 78)	**TAM + HESr** (n = 67)	**p value**
**IPSS total***	8.2 (4.9)	7.8 (5.4)	10.5 (5.2)	0.005
**BII total***	3.2 (2.5)	2.8 (3.0)	4.0 (2.5)	0.023
**IPSS storage sub-score***	3.3 (2.4)	3.2 (2.4)	4.4 (2.5)	0.005
**IPSS voiding sub-score***	4.9 (3.1)	4.6 (3.3)	6.1 (3.2)	0.017
**IPSS item 8 (QoL)***	1.7 (1.2)	1.4 (1.4)	2.2 (1.0)	<0.001

PP: per protocol; HESr: hexanic extract of *Serenoa repens;* TAM: tamsulosin;
IPSS: International Prostate Symptom Score; BII: Benign Prostatic Hyperplasia Impact Index
* Mean (SD)

Table S8. Change from baseline to 6-month follow-up in Qmax and PSA for the three study groups, all patients (ANOVA, PP analysis).

	**TAM**		**HESr**		**TAM + HESr**		**p value**
	**n***	**mean (SD)**	**n***	**mean (SD)**	**n***	**mean (SD)**	
**Qmax (ml/s)**	33	2.8 (4.0)	43	3.3 (4.2)	46	2.1 (3.0)	0.253
**PSA (ng/ml)**	79	0.0 (1.5)	69	-0.1 (0.7)	69	-0.5 (1.1)	0.025

PP: per protocol; HESr: hexanic extract of *Serenoa repens;* TAM: tamsulosin.
Qmax: maximum urinary flow; PSA: prostate-specific antigen
* Number of patients vary according to the test and the personal clinical practice of the investigators.

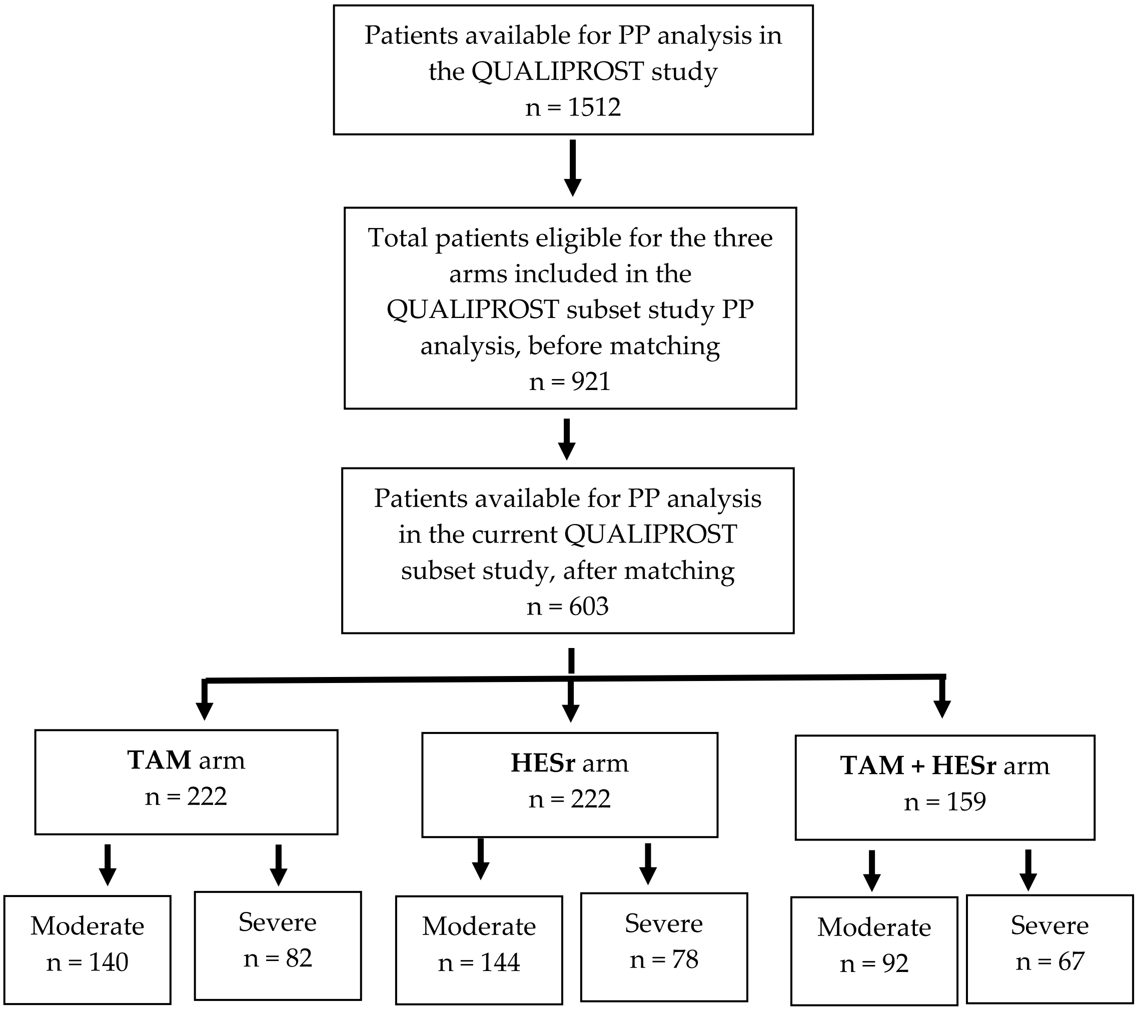

Figure S1. Study flow-chart (PP sample).

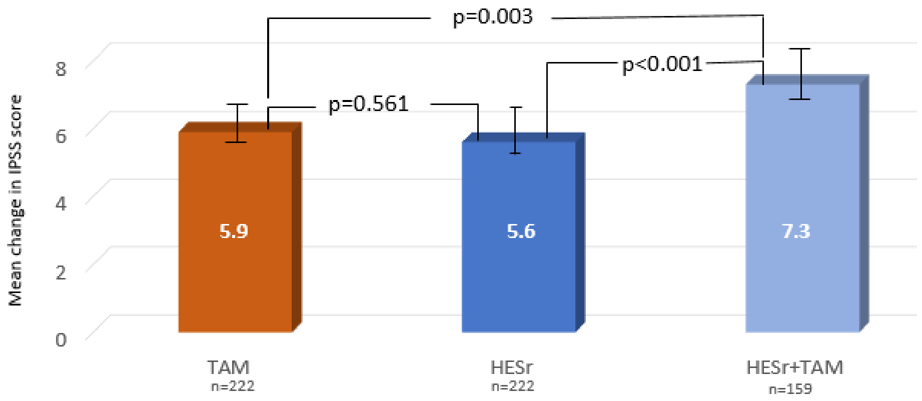

Figure S2. Mean improvement (95% CI) in IPSS total score from baseline to 6 months for the three treatment groups (PP analysis).
IPSS: International Prostate Symptom Score; TAM: tamsulosin; HESr: hexanic extract of *Serenoa repens*.
Student's t-test

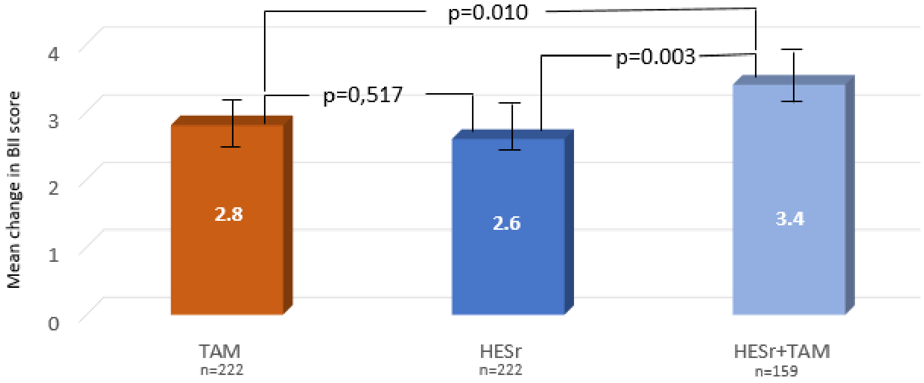

Figure S3. Mean improvement (95% CI) in BII total score from baseline to 6 months for the three treatment groups (PP analysis).
BII: BPH impact index; TAM: tamsulosin; HESr: hexanic extract of *Serenoa repens.* Student's t-test
